# A Case of Superior Mesenteric Venous Thrombosis Managed by Thrombectomy without Bowel Resection

**DOI:** 10.3400/avd.cr.20-00119

**Published:** 2020-12-25

**Authors:** Wakiko Hiranuma, Takuya Shimizu, Miki Takeda, Takayuki Matsuoka, Tadanori Minagawa, Toshiaki Fukutomi, Masato Ohara, Shunsuke Kawamoto

**Affiliations:** 1 Department of Cardiovascular Surgery, Tohoku Medical and Pharmaceutical University, Sendai, Miyagi, Japan; 2 Department of Gastroenterological Surgery, Graduate School of Medicine, Tohoku University, Sendai, Miyagi, Japan; 3 Department of Surgery, Ishinomaki Red Cross Hospital, Ishinomaki, Miyagi, Japan

**Keywords:** superior mesenteric venous thrombosis, laparotomy, thrombectomy

## Abstract

We present a case of superior mesenteric venous thrombosis (SMVT) treated successfully with thrombectomy without bowel resection. A 73-year-old female was referred to our hospital with complaints of stomach ache. The patient was diagnosed with SMVT with impending bowel necrosis and underwent an emergency operation, after computed tomography (CT) revealed a thrombus in the superior mesenteric vein (SMV) extending to the splenic vein, ascites, and extremely edematous intestines. The intestines were not necrotic though highly congested. To avoid massive bowel resection, aggressive thrombectomy was performed. Postoperative CT confirmed resolved SMV and improved bowel edema. Prompt thrombectomy should be considered in such cases.

## Introduction

Superior mesenteric venous thrombosis (SMVT) is an uncommon, critical disease accounting for 6%–15% of mesenteric ischemic diseases, and with a mortality rate of 8%–17%, which reportedly reaches as high as 33% in recurrent cases.^1–4)^ Here we report a case of SMVT in a 73-year-old woman, which was successfully treated by undertaking early thrombectomy without bowel resection.

## Case Report

**Figure 1:**
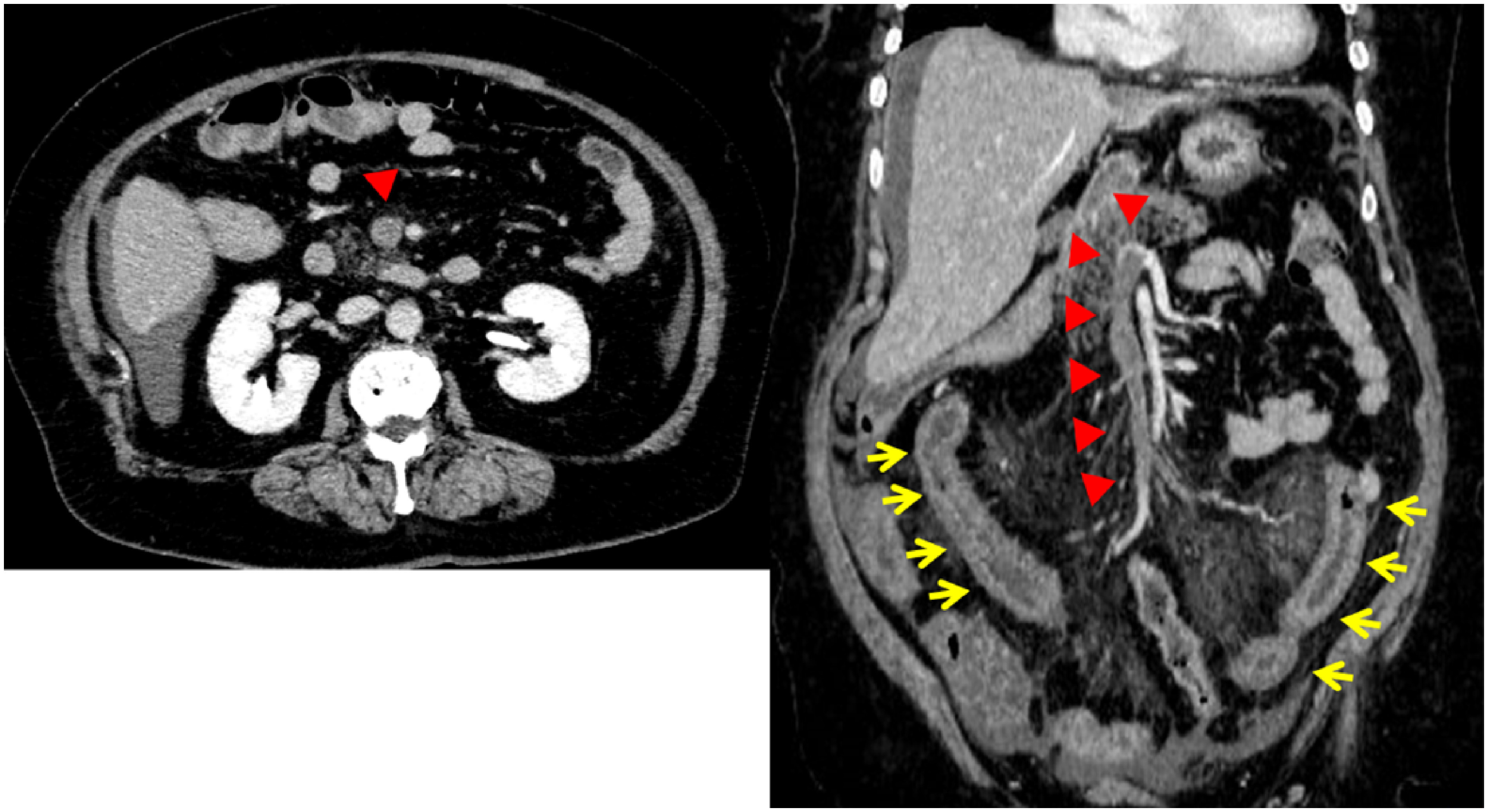
Fig. 1 Preoperative computed tomography of the abdomen and pelvis. A thrombus in the superior mesenteric vein extending to the splenic vein (arrowheads) is noticed along with edematous ileum and ascending colon (arrows).

A 73-year-old woman with a history of diabetes mellitus, dyslipidemia, and Sjogren’s syndrome was referred to our hospital with complaints of abdominal pain and emesis for two days. She also reported a history of ischemic colitis secondary to inferior mesenteric venous thrombosis (IMVT), which had been conservatively treated two years earlier with seven months of warfarin therapy. Physical examination on admission revealed abdominal tenderness and defense. Laboratory data indicated lactic acidosis, and D-dimer level was as high as 50.27 μg/mL. No abnormalities in coagulation factors, or autoantibodies were identified, except Sjogren’s syndrome. Enhanced computed tomography (CT) revealed totally occluded superior mesenteric vein (SMV) with a large thrombus, ascites, and severe edema along the terminal ileum to the ascending colon (**Fig. 1**). We diagnosed her with SMVT and suspected impending bowel necrosis, which required an emergency operation.

**Figure 2:**
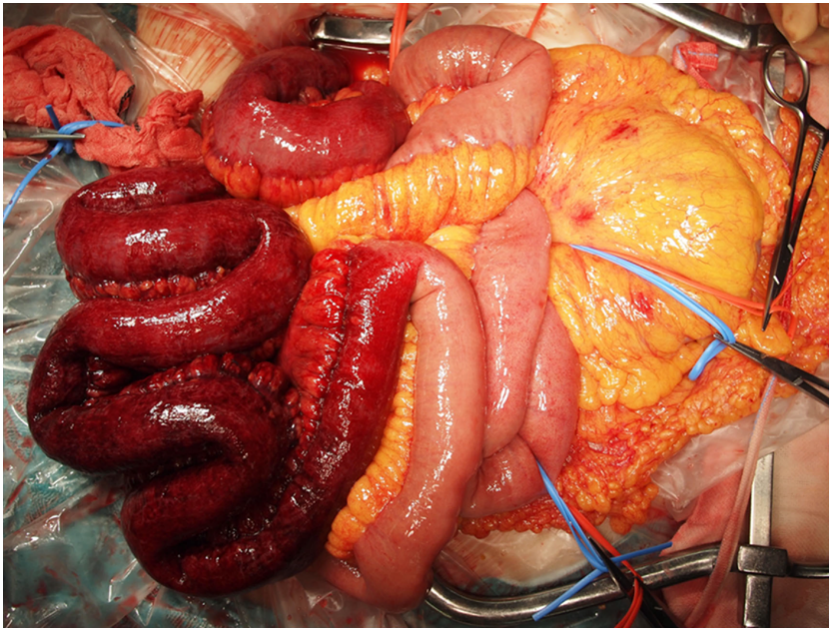
Fig. 2 Intraoperative photograph of the surgical site. The ileum and ascending colon appear extremely edematous and congested. A light hemorrhage is observed in the jejunal mesentery, though bowels are not necrotic.

Laparotomy was performed via a midline approach. A moderate amount of serous ascites was observed. The intestines appeared highly edematous and congested, but it was not apparent that they were actually necrotic (**Fig. 2**). We exposed the SMV and controlled the inferior mesenteric vein, right colic vein, ileocolic vein, and jejunal vein. Anatomically, no anomalies were observed in the portal vein system. A long-axis incision approximately 2-cm long was made in the SMV trunk, and the thrombus was directly removed. In addition, an 8-Fr venous thrombectomy catheter (Edwards Lifesciences LLC, Irvine, CA, USA) was used to remove as much of the residual thrombus as possible, both proximally and distally from the incision site. Then, the incision line was sutured closed. Doppler ultrasonography confirmed blood flow restoration in the SMV. Finally, the abdomen was closed and carefully observed just in case bowel necrosis necessitated a second-look operation.

**Figure 3:**
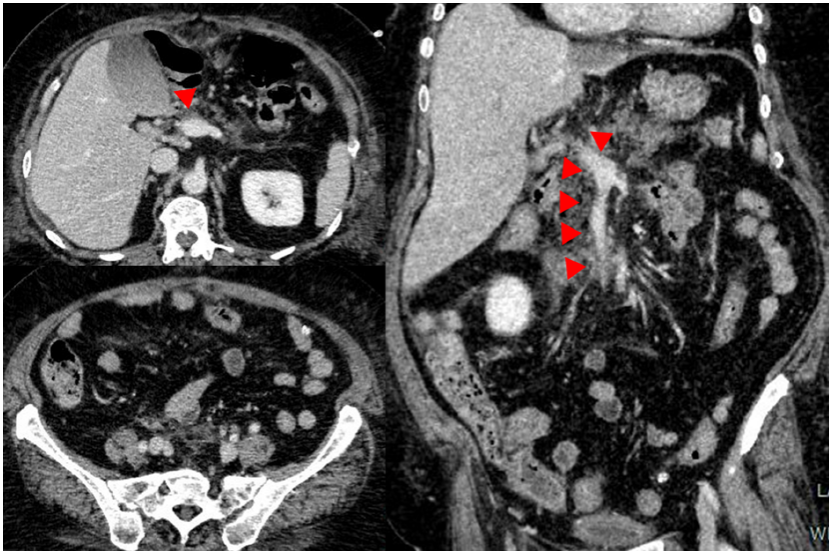
Fig. 3 Postoperative computed tomography of the abdomen and pelvis. The superior mesenteric venous thrombi is resolved (arrowheads), and the edematous bowels have improved.

Anticoagulant therapy with heparin was initiated immediately after the diagnosis, and we strictly controlled it by monitoring activated partial thromboplastin time after the surgery. Intensive care was performed monitoring vital signs, physical examination of abdomen, blood gas analysis, blood tests, and X-ray tests. We comprehensively judged these results which did not indicate bowel necrosis, eliminating the likelihood of a second-look operation. Enhanced CT on postoperative day (POD) 5 demonstrated a patent, thrombus-free SMV and ameliorated bowel edema (**Fig. 3**). Enteral nutrition was started on POD 7. Because the patient was obese as her body mass index was 30.1, it took 18 days to extubate her. Next, the anticoagulant therapy was switched from heparin to warfarin. Eventually, she recovered uneventfully and was transferred to a rehabilitation hospital on POD 35.

## Discussion

SMVT is an uncommon disease with unspecific symptoms and causes an acute abdomen. In most cases, it progresses so slowly that early diagnosis is difficult.^5)^ The highest incidence of SMVT has been reported in people aged 70–79 years, with no gender difference.^2,6)^ With SMVT caused by numerous factors, including cirrhosis, portal hypertension, abdominal surgery, intraperitoneal infection, inflammatory bowel diseases, trauma, hypercoagulability (due to malignancies, pregnancy, and collagen diseases), and hemostatic abnormalities (e.g., protein C or S deficiency, antithrombin III deficiency, and plasminogen abnormality),^2,6)^ it is classified as either idiopathic or secondary, with the latter being the case in about 80% of patients.^7,8)^ In the case reported here, however, these factors were not present. Except the patient had a history of Sjogren’s syndrome whose relationship with SMVT is not yet known. Nonetheless, the patient had a history of recurrent thrombosis due to an uncertain cause.

There exist no clear guidelines in Japan regarding the management of SMVT; however, the European Society of Vascular Surgery (ESVS) has published guidelines on SMVT management in European countries, suggesting that prompt anticoagulant therapy with heparin should be initiated as the first-line treatment immediately after diagnosis.^6)^ If clinical symptoms do not improve even after anticoagulant therapy, interventional radiology (IVR) procedures, such as trans-superior mesenteric arterial thrombolysis and percutaneous transhepatic thrombolysis, are considered. As recommended by the ESVS guidelines, compared with surgery, IVR procedures might have poor therapeutic effects on such totally occluded veins as those observed in the present case; moreover, the desired effects take a certain period of time to occur, resulting in bowel necrosis. Also, the potential risk of bleeding remains a serious concern. Nevertheless they are less invasive and lead to more consistent outcomes.^6)^

It is clear that emergency surgery is indicated if bowel necrosis is suspected at the time of diagnosis. The aims of laparotomy are to evaluate necrotic intestines and relieve venous congestion. In each case, the necessity and site of bowel resection should be carefully considered. Ischemic changes due to SMVT are different from those caused by arterial perfusion insufficiencies, such as superior mesenteric arterial thrombosis, where the affected segments of the intestines appear dark purple in color as a result of congestion; therefore, at present, there is no established way to quantitatively determine the necrotic intestinal segment to be resected and it might be difficult to visually identify the presence or site of necrosis. Bowel anastomosis in cases of highly edematous intestines or microthrombosis might cause anastomotic leakage, whereas maintaining a safety margin may result in excessive bowel resection. Therefore, it has been reported that minimal bowel resection with a second-look operation in mind helps avoid these problems.^5,9)^

Permanent anticoagulant therapy for SMVT is recommended unless contraindicated.^6)^ However, in the present case, anticoagulant therapy with warfarin after ischemic colitis due to IMVT was terminated seven months after the onset of IMVT. We prescribed permanent anticoagulant therapy for this idiopathic SMVT as, although the patient’s local physician confirmed that the thrombus had been resolved at the time of warfarin termination, the patient experienced another ischemic event due to SMVT. We chose warfarin, not direct oral anticoagulants, as anticoagulant therapy for this patient because the recurrence of mesenteric thrombosis occurred after termination of warfarin, which potentially means warfarin was effective, and also favorable in cost.

Recently, there has been a considerable improvement in the mortality rates of patients with SMVT,^2)^ and, with enhanced CT being employed to confirm SMVT in our case, CT being regarded as the most standard and useful tool for the diagnosis of SMVT,^6,10)^ this improvement seems to have resulted from early diagnosis. This is due to the development and availability of CT imaging, as well as from the emergence of various treatment options.

## Conclusion

This study highlighted the overriding importance of prompt diagnosis and immediate laparotomy, especially in cases with a large SMVT and severe bowel congestion, to avoid massive bowel resection, where we dealt with a case of severe SMVT accompanied by impending bowel necrosis, which was successfully managed by prompt thrombectomy without bowel resection. The potential role of permanent anticoagulant therapy in the prevention of recurrent SMVT was also emphasized.
